# OA07 A case of severe osteoporosis treated with teriparatide following the development of bilateral atypical femoral fractures due to IV zoledronic acid

**DOI:** 10.1093/rap/rkad070.007

**Published:** 2023-09-27

**Authors:** Chamith Rosa, Joyal Joseph, Kaushik Chaudhuri

**Affiliations:** University Hospitals Coventry and Warwickshire NHS Trust, Coventry, United Kingdom; University Hospitals Coventry and Warwickshire NHS Trust, Coventry, United Kingdom; University Hospitals Coventry and Warwickshire NHS Trust, Coventry, United Kingdom

## Abstract

**Introduction:**

Atypical femoral fractures (AFF) are a rare complication of long-term anti-resorptive therapy. A literature review of 310 cases of AFF did reveal a median duration of bisphosphonate therapy of 7 years. Additionally, simultaneous glucocorticoid use was seen in 34% of the cases and was reported to have a 5-fold increase in one case series. Around 70% of patients do describe a prodrome of thigh pain and around quarter of all cases will have bilateral involvement.

**Case description:**

A 59-year-old lady with a background diagnosis of Behçet’s disease was referred for continuation of osteoporosis care.

**History of Fractures:**

2003- left-sided distal radial fracture; 2017 - refractured again after a fall; left 5th metatarsal fracture a few months later. 2019 - right navicular fracture, left pubic rami fracture and left iliac wing fracture, after a fall from the bus

In 2023, she has tripped and fell from the standing height resulting in a left femoral shaft fracture, which had radiological features of an atypical femoral fracture. She denied any prodromal thigh pain. The orthopaedic team did an intramedullary nailing to the left femoral shaft. X-ray of the right femur shaft revealed cortical thickening of right mid femoral shaft.

**Treatment History:**

In 2009, she was started on oral bisphosphonate therapy and from 2020 to 2022 received 3 doses of IV zoledronic acid at the national Behçet’s centre in Birmingham. She has been on long-term steroid therapy due to the underlying Behçet's disease and was on prednisolone 7mg daily when she was referred to us.

**Management of AFF:**

The orthopaedic team initially recommended a watch and wait policy for the right side; however, following discussions with the patient and with us, prophylactic nailing of the right femoral shaft was done 2 months later.

**Investigations:**

DXA scan in 2022: spine T-score of−3.3 (L2 −3.6, L3 −3.9, L4 −3.7); femoral neck T-score of −2.5. Despite treatment with oral and then parenteral bisphosphonates for the last 12 years, there is a significant reduction in BMD with 18.2% decrease in the spine and 7.1% decrease in the femoral neck compared to the last DEXA in 2019.

**Management plan:**

The next step was to treat her with teriparatide, which she is currently having without any problems. We shall continue it for 24 months.

**Discussion:**

Atypical femoral fractures are rare. Anti-resorptive medication including bisphosphonates and denosumab are known to cause atypical femoral fractures. Teriparatide, being anabolic agent, is not associated with the increased risk of atypical femoral fractures. While there are no randomised controlled studies about the use of teriparatide in healing atypical femoral fractures, there are some case reports where teriparatide had shown improved fracture healing in AFF.

After the 24 months, to ‘lock in the effects’ of teriparatide, an anti-resorptive medication will be required. Because both femoral shafts have received intramedullary nailing, we would consider IV zoledronic acid after the 24 months of teriparatide. This is the approach supported by the European Calcified Tissue Society in their systemic review and recommendations on ‘Medical Management of Patients After Atypical Femur Fractures’ (https://doi.org/10.1210/clinem/dgz295).

**Key learning points:**

Consider atypical femoral fracture in patients who are on anti-resorptive therapy and complain of thigh/groin pain.

Atypical femoral fractures can be bilateral; therefore, evaluate both sides.

Teriparatide can be used to treat osteoporosis in patients with atypical femoral fracture, but considerations will have to be made in relation to subsequent therapy.

